# Spermatocytic tumor of the testis: A case report and mini‑review of the literature

**DOI:** 10.3892/mi.2023.111

**Published:** 2023-09-11

**Authors:** Rawa Bapir, Ismaeel Aghaways, Rawa M. Ali, Saman S. Fakhralddin, Rebaz M. Ali, Rezheen J. Rashid, Ari M. Abdullah, Muhammed Bag A. Ali, Karukh K. Mohammed, Hiwa O. Abdullah, Fahmi H. Kakamad

**Affiliations:** 1 Department of Scientific Affairs, Smart Health Tower, Sulaimani, Kurdistan 46001, Iraq; 2 Department of Urology, Sulaimani Teaching Hospital, Sulaimani, Kurdistan 46001, Iraq; 3 Kscien Organization for Scientific Research, Sulaimani, Kurdistan 46001, Iraq; 4 College of Medicine, University of Sulaimani, Sulaimani, Kurdistan 46001, Iraq; 5 Department of Oncology, Hiwa Hospital, Sulaimani, Kurdistan 46001, Iraq; 6 Department of Radiology, Hiwa Hospital, Sulaimani, Kurdistan 46001, Iraq; 7 Department of Pathology, Sulaimani Teaching Hospital, Sulaimani, Kurdistan 46001, Iraq; 8 Smart Health Tower (Raparin branch), Sulaimani, Kurdistan 46001, Iraq

**Keywords:** spermatocytic tumor, testicular cancer, spermatocytic seminoma, orchidectomy

## Abstract

Spermatocytic tumors are a rare type of testicular cancer, comprising <1% of all testicular malignancies. This type of cancer typically affects males in their 60s and 70s and rarely metastasizes; however, it poses a threat to the health of affected individuals if left untreated. The present study describes the case of a 68-year-old male patient with this type of tumor, including a presentation of his initial symptoms, treatment and subsequent monitoring. A male patient, aged 68 years, visited the authors' clinic with an asymptomatic mass in the right testicle. The mass had been progressively increasing in size for a duration of 5 years following a history of blunt injury. During the examination, a noticeable, painless enlargement was detected in the right testis, whereas the left testis appeared to be in a normal state. Tumor markers were within normal limits. Imaging revealed a complex mass (11x8x7 cm) almost replacing the right testis, with no detectable lymph nodes. A right radical orchidectomy was performed under spinal anesthesia. A histopathological examination revealed a spermatocytic tumor. The post-operative period was uneventful, with no metastasis detected in the CT scans. The patient was discharged with instructions for regular follow-up appointments. The case presented herein highlights a rare spermatocytic tumor in a 68-year-old male. The early detection and treatment of testicular tumors, regardless of age, are crucial for a good prognosis.

## Introduction

Testicular cancer is an uncommon neoplasm, contributing to 1.5% of all malignancies in the male population (1). This type of cancer is more commonly found in young males (15-35 years of age); however, it can also manifest in elderly individuals (2,3). Tumors originating in the testicles are classified into germ-cell and non-germ-cell tumors. The overwhelming majority (>95%) stem from germ-cell lineage. Germ cell tumors encompass seminoma, non-seminoma and exceedingly rare spermatocytic tumors (ScTs) (4). The prevalence of germ cell tumors is increasing globally, although notable regional variations are evident. These tumors are exceptionally infrequent in Africa, among Afro-American communities, and in Asia. Conversely, there appears to be a greater occurrence of such tumors in Nordic European nations, particularly in Denmark, Norway and Sweden (4). ScT is a rare form of germ cell tumor, comprising less than 1% of all cases. It is most commonly diagnosed in males in their 60s and 70s. This tumor is slow-growing and typically does not spread to other parts of the body. However, it can still be life-threatening if not diagnosed and treated at an early stage (4,5). It was previously classified as a type of seminoma. However, it is currently recognized as a distinct entity due to several important differences (5). The distinctive clinical characteristics that differentiate ScTs from classical seminoma include presentation at an elderly age, a lack of an undescended testicle and a diminished inclination for metastasis (6). However, there is a minimal risk of recurrence and metastasis, particularly among older patients (7). The appropriate management of individuals with ScTs has not yet been firmly established due to their exceptionally low occurrence. Radical orchiectomy is recommended by some experts, while others argue that testis-sparing surgery may provide an adequate alternative (5,8).

The present study describes a rare case of a spermatocytic tumor in an elderly male, including presentation, investigations, management and follow-up. The present study aimed to avoid citing predatory publications based on a well-known predatory list (9).

## Case report

### Patient information

A 68-year-old male patient presented to the Urology Clinic at Smart Health Tower, reporting a gradually enlarging, painless right testicular mass for a period of 5 years. The case had a history of right testicular blunt trauma 5 years prior. He had ignored his condition until the mass grew to a size that began to concern him.

### Clinical findings

Upon a physical examination, it was found that he had an enlarged, non-tender right testicular mass with no associated palpable inguinal lymph nodes, and the left testis appeared normal.

### Diagnostic assessment

**Figure 1:**
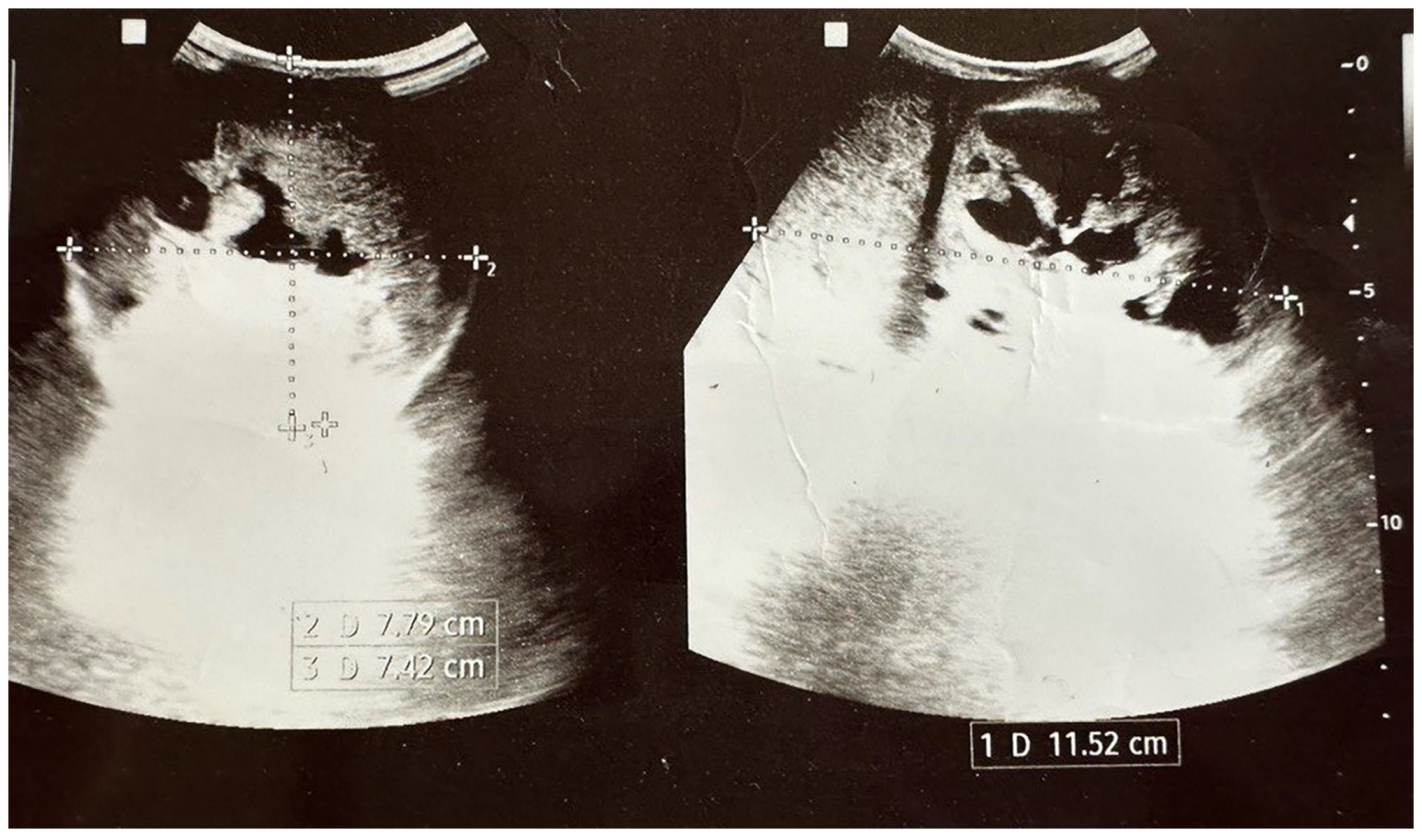
Two orthogonal planes of ultrasound images illustrating a large, well-circumscribed, solid mass measuring 11x7x8 cm, completely replacing the right testicle. The lesion exhibited multifocal cystic degeneration resulting in posterior acoustic enhancement.

The levels of tumor markers, including alpha-fetoprotein (AFP; 1.81 ng/ml; normal range, 0-40 ng/ml) and human chorionic gonadotropin (hCG; 0.448 µIU/ml; normal range, <3 µIU/ml) were within normal limits, while those of lactate dehydrogenase were elevated to 400.3 U/l (normal range, 135-225 U/l). Scrotal ultrasound (US) imaging revealed a complex heterogeneous mass almost replacing the right testis and measuring 11x8x7 cm (Fig. 1), while the left testis appeared normal. No para-aortic lymph nodes were detected on an abdominal US scan.

### Therapeutic intervention

**Figure 2:**
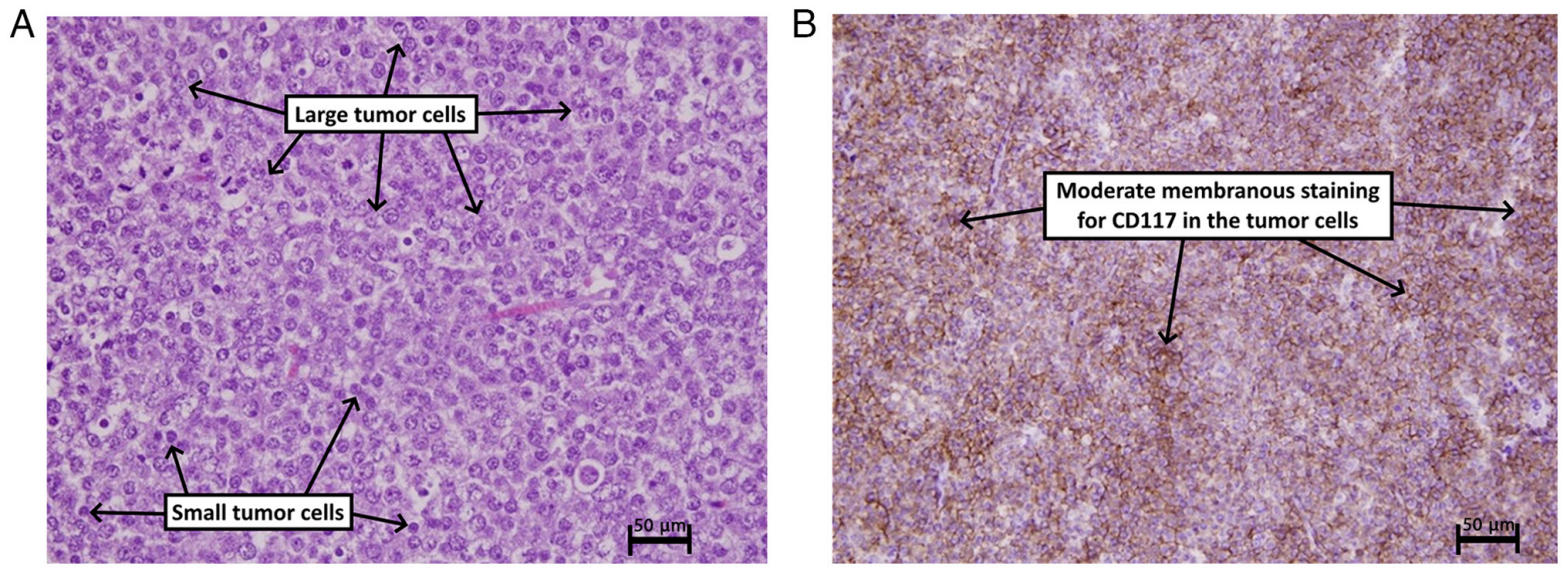
(A) The section illustrates sheets of variably sized cells, including small cells with scant cytoplasm and round hyperchromatic nuclei and large cells with a moderate amphophilic cytoplasm and round vesicular nuclei. There is notable mitotic activity and no intratubular germ cell neoplasia (hematoxylin and eosin staining, x400 magnification). (B) The section illustrates diffuse, moderate membranous reactivity of the tumor cells for the CD117 antibody (immunohistochemistry for CD117 antibody with diaminobenzidine chromogen, x100 magnification).

Under spinal anesthesia, a right radical orchidectomy was performed. Histopathological and immunohistochemical analysis revealed the presence of a spermatocytic tumor in the testis (Fig. 2).

The histopathological examination was performed by the authors' laboratory as follows: The sections (5-µm-thick) were paraffin-embedded and fixed with 10% neutral buffered formalin at room temperature for 24 h. They were then stained with hematoxylin and eosin (Bio Optica Co.) for 1-2 min at room temperature, and examined under a light microscope (Leica Microsystems GmbH).

For immunohistochemistry, the sections (4-6-µm-thick) were cut from the paraffin blocks and transferred onto glass slides with an electric charge. These slides were then placed in an oven at 60˚C overnight. Antigen retrieval was carried out using the Dako PT Link (Agilent Technologies, Inc.) by immersing the sections in boiling water at 100˚C for 5 to 10 min. Depending on the target antibody, a solution with either pH 6.0 or 9.0 was used. Following antigen retrieval, the slides were washed for 15 min with a 20 ml buffer solution (0.05 mol/l Tris/HCl, 0.15 mol/l NaCl, 0.05% Tween-20, pH 7.6) at room temperature. To facilitate this process, the slides were marked using the Dako Pen (Agilent Technologies, Inc.). Endogenous peroxidase activity was blocked by applying a 3% hydrogen peroxide solution. Subsequently, primary antibodies were applied at room temperature followed by incubation at room temperature (25˚C) for 80 min. Afterward, the secondary antibody, which was horseradish peroxidase, was applied, along with the chromogen (diaminobenzidine), both at room temperature for 15 min. To achieve counterstaining, hematoxylin Gill II was applied at room temperature for 30 sec. Finally, the slides were allowed to dry, and coverslips were affixed.

### Follow-up

The post-operative period was uneventful, and the metastatic workup, including chest and abdominal computed tomography scans, revealed no evidence of metastasis. The patient was discharged the day after the surgery and ws instructed to return for regular follow-up appointments.

## Discussion

Continual discussions exist regarding the theory that suggests precursor cells of ScTs arise during embryogenesis (10). Certain scholars, however, hold the view that ScTs emerge from fully matured cells such as pachytene spermatocytes (5). In addition, both the morphological and immunohistochemical attributes of the tumor cells have indicated an origin from spermatogonial stem cells (11). These tumors originate from primary spermatocytes that have progressed to at least the initiation stage of prophase meiosis (5). They are characterized by a distinctive amplification of chromosome 9, which corresponds to the DMRT1 gene and are consistently devoid of any association with other forms of germ cell tumors (5).

ScTs are rare, accounting for <1% of all testicular cancers. These type of tumors predominantly affect older males, typically in their 60s and 70s (7). However, previous studies have reported the disease in young males aged <40 years and suggested for clinicians and pathologists to be cognizant of the fact that ScTs can manifest even in young patients. In a systematic review of 146 cases performed by Grogg *et al* (5), it was revealed that the majority of the cases presented with testicular pain and/or enlargement. In addition, the tumor in several cases was diagnosed after workup for other concerns, a such as infertility, hydrocele, metastasis, weight loss and back pain (5). They also reported that the tumor caused no changes in the levels of testosterone, estrogen or the clinical features of gynecomastia (5). In the case presented herein, the gradual enlargement of the painless testicular mass over a 5-year period is consistent with the indolent nature of ScTs. The patient's history of blunt testicular trauma 5 years prior to presentation raises the possibility of a causal relationship, although the exact etiology of ScTs remains uncertain (4,7). It is worth noting that the patient neglected his condition until the mass grew to a size that began to concern him. This delay in seeking medical attention underscores the importance of raising awareness about testicular health and the significance of early detection. In a previous case series study, Rabade *et al* (8) reported that unilateral testicular masses were evident in all patients. Instances of bilateral involvement have been infrequently documented, primarily manifesting as metachronous tumors. The range of tumor size has been diverse, spanning from 2 to 20 cm, with an average measurement of 7 cm (8). A clinical examination plays a crucial role in the evaluation of testicular masses. In the case in the present study, the non-tender, enlarged right testicular mass (11 cm) with no associated palpable inguinal lymph nodes raised the suspicion of a testicular tumor. The contralateral testis appeared normal, which is consistent with the unilateral nature of ScTs (1,3,4,7,8). The levels of tumor markers, such as AFP and hCG were within the normal range, which is consistent with the diagnosis of ScT (1,12). It should be noted that these tumor markers are typically elevated in other types of testicular cancers, such as yolk sac tumor and choriocarcinoma (12).

In the case presented herein, scrotal US imaging revealed a complex heterogeneous mass almost replacing the right testis, confirming the presence of a sizable tumor. The absence of para-aortic lymph nodes on abdominal ultrasound scan suggested localized disease without metastasis (13,14). Another study on managing 21 cases reported no metastasis (8). Although, other scholars have reported 14 cases of metastasis out of 146 cases, and ScTs with other histological variants such as sarcoma and anaplastic tumors tend to metastasize more than the pure ScTs (5,8). Despite the absence of trials regarding the management of ScTs, however, orchiectomy remains the standard treatment option based on the reports and clinical series (5). Testis sparing surgery is not recommended due to the difficulty of differentiating ScTs from pure seminoma, in some cases even in frozen section analysis (5). The case in the present study underwent radical orchiectomy, which allows for accurate staging and histopathological examination of the excised specimen (5). The differential diagnoses of ScTs in histopathological or immunohistochemistry can be classical seminoma, embryonal cancer and non-Hodgkin lymphoma. Hence, it is recommended for pathologists to be firmly aware of the distinctive features of each disease (8). In the case in the present study, histopathological and immunohistochemical analyses confirmed the diagnosis of a spermatocytic tumor. Post-operatively, the patient had an uneventful recovery. Metastatic workup, including chest and abdominal CT scans, revealed no evidence of metastasis. Regular follow-up appointments were scheduled to monitor the patient's condition and detect any potential recurrence or metastasis. Long-term surveillance is crucial due to the risk of late recurrence, even though these tumors tend to exhibit a slow and indolent course (15,16).

In conclusion, the present case report underscores the rarity and slow-growing nature of testicular ScTs, emphasizing the significance of prompt identification and treatment. Clinical examination, tumor markers, and imaging techniques are vital for accurate diagnosis. Radical orchidectomy is crucial for ensuring favorable outcomes, and long-term monitoring is necessary to detect any potential recurrence or spread.

## Data Availability

The datasets used and/or analyzed during the current study are available from the corresponding author on reasonable request.

## References

[1] Facchini G, Rossetti S, Berretta M, Cavaliere C, D'Aniello C, Iovane G, Mollo G, Capasso M, Della Pepa C, Pesce L (2019). Prognostic and predictive factors in testicular cancer. Eur Rev Med Pharmacol Sci.

[2] Mahmood ZH, Mohemed FM, Fatih BN, Qadir AA, Abdalla SH (2023). Cancer publications in one year (2022); a cross-sectional study. Barw Med J.

[3] Williamson SR, Delahunt B, Magi-Galluzzi C, Algaba F, Egevad L, Ulbright TM, Tickoo SK, Srigley JR, Epstein JI, Berney DM (2017). The World Health Organization 2016 classification of testicular germ cell tumours: A review and update from the international society of urological pathology testis consultation panel. Histopathology.

[4] Secondino S, Rosti G, Tralongo AC, Nolè F, Alaimo D, Carminati O, Naspro RLJ, Pedrazzoli P (2022). Testicular tumors in the ‘elderly’ population. Front Oncol.

[5] Grogg JB, Schneider K, Bode PK, Wettstein MS, Kranzbühler B, Eberli D, Sulser T, Beyer J, Hermanns T, Fankhauser CD (2019). A systematic review of treatment outcomes in localised and metastatic spermatocytic tumors of the testis. J Cancer Res Clin Oncol.

[6] Pendlebury S, Horwich A, Dearnaley DP, Nicholls J, Fisher C (1996). Spermatocytic seminoma: A clinicopathological review of ten patients. Clin Oncol (R Coll Radiol).

[7] Colecchia M, Bertolotti A

[8] Rabade K, Panjwani PK, Menon S, Prakash G, Pal M, Bakshi G, Desai S (2022). Spermatocytic tumor of testis: A case series of 26 cases elucidating unusual patterns with diagnostic and treatment dilemmas. J Cancer Res Ther.

[9] Muhialdeen AS, Ahmed JO, Baba HO, Abdullah IY, Hassan HA, Najar KA, Mikael TM, Mustafa MQ, Mohammed DA, Omer DA (2023). Kscien's list; A new strategy to discourage predatory journals and publishers (second version). Barw Med J.

[10] Menon S, Karpate A, Desai S (2009). Spermatocytic seminoma with rhabdomyosarcomatous differentiation: A case report with a review of the literature. J Cancer Res Ther.

[11] Waheeb R, Hofmann MC (2011). Human spermatogonial stem cells: A possible origin for spermatocytic seminoma. Int J Androl.

[12] Pedrazzoli P, Rosti G, Soresini E, Ciani S, Secondino S (2021). Serum tumour markers in germ cell tumours: From diagnosis to cure. Crit Rev Oncol Hematol.

[13] Marko J, Wolfman DJ, Aubin AL, Sesterhenn IA (2017). Testicular seminoma and its mimics: From the radiologic pathology archives. Radiographics.

[14] Xue N, Wang G, Zhang S, Lu Y (2023). The value of contrast-enhanced ultrasonography in differential diagnosis of primary testicular germ cell tumors and non-germ cell tumors over 50 years old. Front Oncol.

[15] Ruf CG, Schmidt S, Kliesch S, Oing C, Pfister D, Busch J, Heinzelbecker J, Winter C, Zengerling F, Albers P (2022). Testicular germ cell tumours' clinical stage I: Comparison of surveillance with adjuvant treatment strategies regarding recurrence rates and overall survival-a systematic review. World J Urol.

[16] Stoop H, van Gurp R, de Krijger R, Geurts van Kessel A, Köberle B, Oosterhuis W, Looijenga L (2001). Reactivity of germ cell maturation stage-specific markers in spermatocytic seminoma: Diagnostic and etiological implications. Lab Invest.

